# Supplementary material for: Deletion of wfs1 Impairs Oligodendrocyte Precursor Cells Dorsal Distribution and Myelination Through the wfs1-hmgcs1 Axis in Zebrafish
Source: Biology (Basel). 2026 Aug 16;15(16):1402. doi: 10.3390/biology15161402 (PMC13510151; doi:10.3390/biology15161402)
Supplement: Supplementary file 1 [file biology-15-01402-s001.zip › File S1-Transcriptome sequencing data and differentially expressed genes (DEGs) in wfs1-- zebrafish at 5 dpf/2.KEGG/wfs1KOvsWT/ALL/src/dre03022.html]

dre03022


- dre:798997

- Up regulated genes

798997\_1(8.66470635883548) 798997(4.55463031105369)

- Down regulated genes

798997\_2(-3.06436893863248)

- dre:567899

- Down regulated genes

567899(-1.51323151491026)

Close
